# Association among raised intraventricular pressure, clinical signs, and magnetic resonance imaging findings in dogs with congenital internal hydrocephalus

**DOI:** 10.1111/jvim.17235

**Published:** 2024-10-31

**Authors:** Daniela Farke, Agnieszka Olszewska, Kathrin Büttner, Martin J. Schmidt

**Affiliations:** ^1^ Department of Veterinary Clinical Sciences, Small Animal Clinic Justus‐Liebig‐University, Frankfurter Strasse 108 Giessen 35392 Germany; ^2^ Unit for Biomathematics and Data Processing, Faculty of Veterinary Medicine Justus Liebig‐University‐Giessen Giessen Germany

**Keywords:** hydrocephalus, intraventricular pressure, MRI

## Abstract

**Background:**

Dogs with internal hydrocephalus do not necessarily have high intraventricular pressure (IVP).

**Hypothesis/Objectives:**

Not all reported MRI findings indicate high IVP and some clinical signs might be associated with elevated IVP and syringomyelia.

**Animals:**

Fifty‐three dogs.

**Materials and Methods:**

Cross‐sectional study. Clinical signs and MRI findings were evaluated for an association of IVP >12 mm Hg and syringomyelia.

**Results:**

High IVP was associated with obtundation OR 4.64 (95% CI 1.27‐16.93) (*P* = .02), head tilt OR 6.42 (95% CI 1.08‐37.97) (*P* = .04) and nystagmus OR 8.24 (95% CI 1.44‐47.07) (*P* = .02). Pain was associated with syringomyelia OR 3.4 (95% CI 0.98‐11.78) (*P* = .05). The number of affected ventricles was associated with high IVP OR 2.85 (95% CI 0.97‐8.33) (*P* = .05) and syringomyelia OR 12.74 (95% CI 2.93‐55.4) (*P* = .0007). Periventricular edema OR 24.46 (95% CI 4.54‐131.77), OR 7.61 (95% CI 1.91‐30.32) (*P* < .0002, *P* = .004) and signal void sign OR 17.34 (95% CI 4.01‐74.95), OR 4.18 (95% CI 1.16‐15.02) (*P* < .0001, *P* = .03) were associated with high IVP and syringomyelia. The probability for syringomyelia is lower with disruption of the internal capsule OR 0.19 (95% CI 0.05‐0.72) (*P* = .01) and higher VBR OR 0.25 (95% CI 0.1‐0.63) (*P* = .004).

**Conclusions and Clinical Importance:**

Previously reported MRI findings are not predictive of high IVP. Clinical signs and MRI findings should be used to make a diagnosis of internal hydrocephalus in dogs with or without high IVP.

## 1 INTRODUCTION

Internal hydrocephalus can be classified based on pathophysiology (primary‐secondary), time of onset (congenital, neonatal, adult), and impeded passage of CSF (obstructive or vs non‐obstructive).^1^, ^2^ Although the term hydrocephalus might imply a high intraventricular pressure (IVP), ventricular distension can occur with normal, subnormal, or high IVP in dogs.^3^ It is likely that IVP is highly dynamic, both in the physiological state as well as during the development of internal hydrocephalus. Values of IVP exceeding reported reference values (5‐12 mm Hg), up to 25 mm Hg, can transiently occur in healthy dogs without clinical signs, depending on the head position.^4^ Based these findings, congenital internal hydrocephalus in dogs might also be classified based on the presence of a high, normal, or even low IVP.^3^ This has important implications for the selection of an appropriate valve within the VPS system, and intraoperative IVP measurement was recommended to measure the individual IVP.^3^

A rapid rise in IVP can lead to serious complications in hydrocephalic humans, including loss of consciousness, and brain herniation.^5^ It might also cause disturbances of the cardiopulmonary control centers in the brainstem, resulting in sudden cardiorespiratory arrest in hydrocephalus patients.^5^, ^6^, ^7^, ^8^, ^9^, ^10^, ^11^ Humans with increased intracranial pressure (ICP) are presented with headache and vision deficits in an early state of the disease.^12^, ^13^ We assume that hydrocephalic dogs with low or normal IVPs have non‐ or at least slowly progressive clinical signs, whereas dogs with high IVPs might be at risk of having rapidly progressive signs, which potentially become life‐threatening, as in humans.^5^, ^6^, ^7^, ^8^, ^9^, ^10^, ^11^ It would be beneficial to have clinical and imaging indicators implying high IVP, especially if diagnosis and treatment do not take place in the same hospital.

Syringomyelia (SM) is reported to be a concurrent finding in animals and humans with internal hydrocephalus.^14^, ^15^, ^16^ Reduced cross‐sectional area of the subarachnoid space and altered CSF hydrodynamics play a role in pathophysiology of SM and disturbances in CSF flow are suspected to result in subsequent central canal dilation.^17^, ^18^

Specific MRI signs were proposed that might indicate a high IVP, but these have not been correlated with IVP measurements.^19^ This study compared previously reported imaging criteria to intraoperative IVP measurements.^19^ We hypothesize that some, but not all previously reported MRI findings truly indicate high IVP and that some clinical signs might be more common in cases of high IVP. We also evaluated if SM is associated with clinical sings or further MRI findings in these animals.

## 2 MATERIALS AND METHODS

In this study, medical record databases of the Department of Veterinary Clinical Science, Small Animal Clinic, Justus‐Liebig‐University Giessen, Germany were searched for records of client owned dogs with a diagnosis of internal hydrocephalus and implantation of a VPS dated from January 2001 to June 2023. Individual dog characteristics were extracted from the database in addition to IVP measurements and an assessment of the presence or absence of SM. Our study design was therefore a cross‐sectional study (with exposure and outcome variables assessed at the same time) but with an extended period of study subject recruitment, from January 2001 to June 2023. Only animals with congenital internal hydrocephalus were selected for the study. This includes communicating and non‐communicating hydrocephalus (eg, in case of aqueductal stenosis). The presence of any acquired cause for internal hydrocephalus like structural neoplastic or inflammatory lesions were defined as exclusion criteria. CSF examination was routinely performed before surgery to further exclude inflammatory disease. Abnormalities were defined as a total nucleated cell count >5 cells/μL and a protein count >300 g/L. Images and medical records were evaluated by a board‐certified neurologist. Data collected from the records included the animal's age in months at the time of diagnosis, breed, sex, body weight, duration of clinical signs, clinical signs, magnetic resonance imaging findings, and IVP measured in surgery. All further mentioned procedures took place in the Department of Veterinary Clinical Science, Small Animal Clinic, Justus‐Liebig‐University Giessen, Germany.

### 2.1 Magnetic resonance imaging

Imaging was performed in the Department of Veterinary Clinical Science, Small Animal Clinic, Justus‐Liebig‐University Giessen, Germany using a 3.0 Tesla high field MRI scanner (Phillips Intera Gyroscan, Philips Healthcare, Hamburg, Germany) or 1.5 Tesla high‐field MRI scanner (Siemens Verio, Siemens Healthcare, Erlangen, Germany). Animals were positioned in sternal recumbency. Images included at least sagittal, transverse, and dorsal T2‐weighted images, transverse Fluid attenuating inversion recovery (FLAIR) sequences, and transverse T1‐weighted pre‐ and post‐gadolinium (Omniscan™ 0.5 mmol/mL) (0.2 mL/kg, IV) contrast medium administered images. Imaging settings for the 1.5 Tesla MRI were performed as following: T2‐weighted images (Turbo Spin Echo, Time of repetition [TR] 4830 ms, Time of echo [TE] 120 ms, slice thickness 3 mm), FLAIR (TE = 96 ms, Time of inversion [TI] = 2499 ms, TR = 9000 ms, slice thickness 2.5 mm) and T1‐weighted images (TR 550 ms, TE 10 ms, slice thickness 1 mm). Imaging settings for the 3. Tesla MRI were performed as following: T2‐weighted images (Turbo Spin Echo, TR = 4290 ms, TE = 120 ms, slice thickness 3 mm), FLAIR (TE = 91 ms, TI = 1980 ms, TR = 5780 ms, slice thickness 2.5 mm) and T1‐weighted images (TR 600 ms, TE 9 ms, slice thickness 1 mm).

### 2.2 Image analysis

**FIGURE 1:**
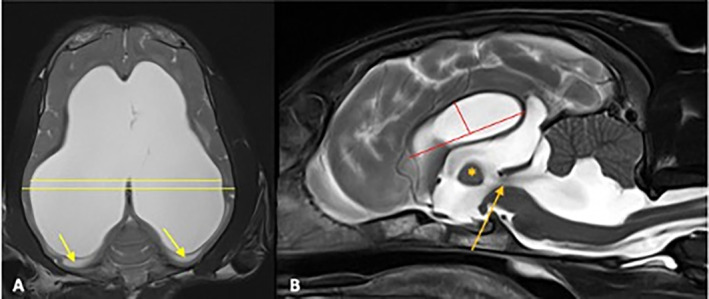
T2‐weighted MR images of the brain of 2 dogs with congenital internal hydrocephalus in a dorsal plane (A) with an IVP of 18 mm Hg and a midsagittal plane (B) with an IVP of 22 mm Hg. The yellow lines indicate measurement of the VBR (A). The red lines show the measurement of the corpus callosal height. A line is drawn from the ventral border of the genu of the corpus callosum to the ventral border of the splenium of the corpus callosum, another tangential line is drawn from that line up to the highest point of the body of the corpus callosum (B). Periventricular edema within the white matter of the occipital lobes is shown in picture A (yellow arrow). A deformed, triangular shaped interthalamic adhesion (asterisk) and signal void sign within the 3rd ventricle, mesencephalic aqueduct and 4th ventricle can be seen in picture B (orange arrow).

**FIGURE 2:**
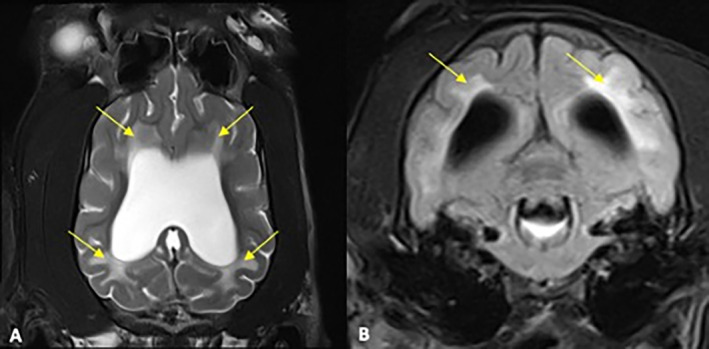
Dorsal, T2‐weighted (A) and transversal FLAIR (B) MR‐images of a dog with an IVP of 22 mm Hg. The arrows point out the periventricular edema within the white matter.

**FIGURE 3:**
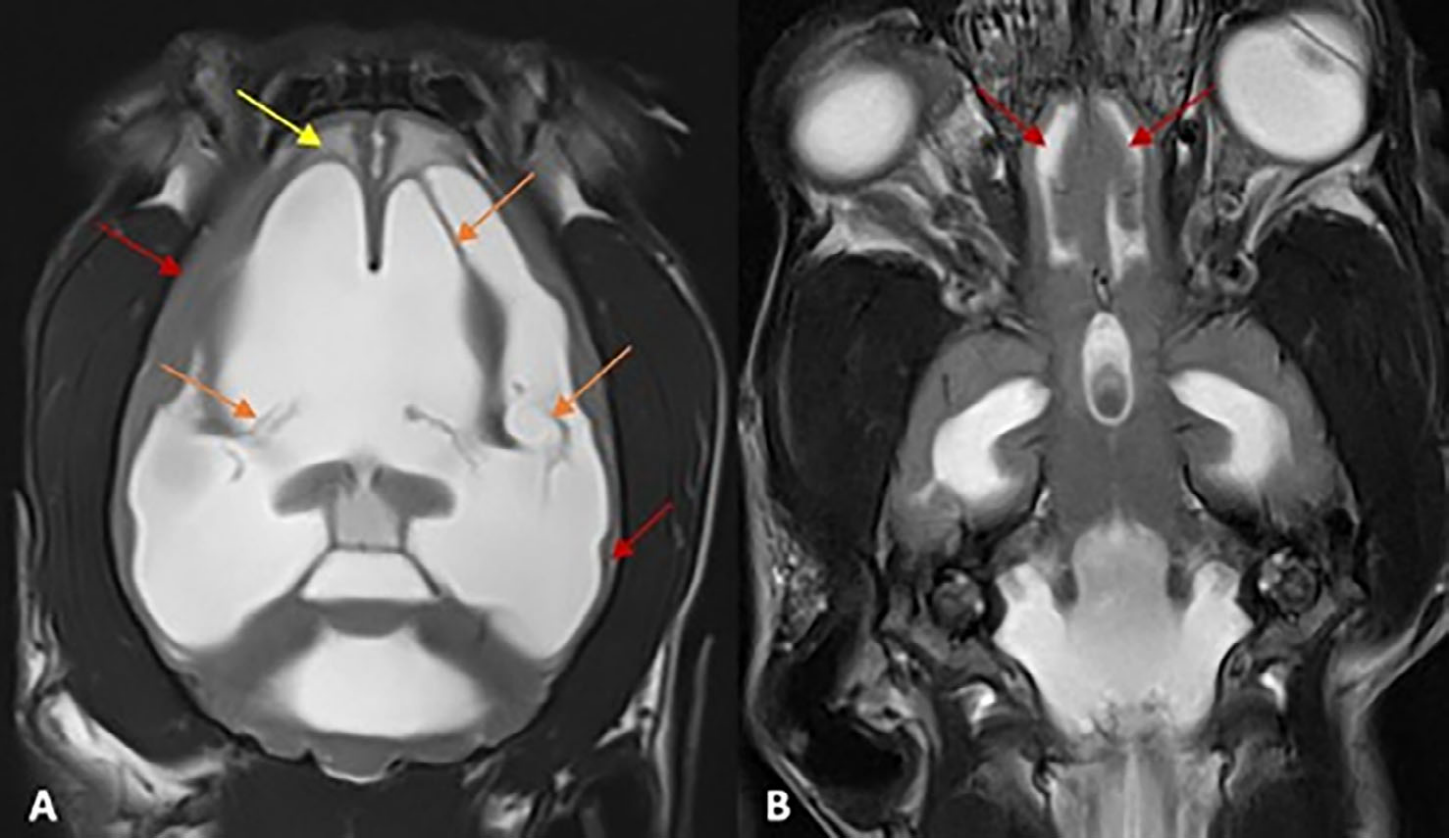
T2‐weighted MR images of the brain of in dorsal plane at the level of the hemispheres of a dog with an IVP of 22 mm Hg (A) and at the level of the olfactory bulbs of a dog with an IVP of 16 mm Hg (B). The yellow arrow points to periventricular edema, the orange arrows show disruptions of the internal capsule, and the red arrows show a flattening of cortical gyri and sulci (A). The dilation of the olfactory recesses is shown in picture B (red arrow).

All MRI datasets were retrieved from the relevant PACS system and evaluated retrospectively by a board‐certified neurologist. The images were evaluated for morphometric criteria (ventricle brain ratio [VBR] and corpus callosal height) (Figure 1) and morphological criteria (deformation of the interthalamic adhesion, periventricular edema, flattening of gyri and sulci, dilation of the olfactory recess, disruption of the internal capsule) as previously described (Figures 2 and 3).^19^ In addition, the presence of a signal void within the 3rd ventricle or mesencephalic aqueduct, the number of affected ventricles, and the presence of SM were assessed (Figure 1).

### 2.3 Anesthesia

A standard anesthetic protocol was used for MRI examination and surgical procedure in each animal. Diazepam (0.5 mg/kg) was administered intravenously into a venous catheter (18, 20, or 22 gauge) placed in the right or left cephalic vein. Anesthesia was induced with propofol (2‐4 mg/kg, IV). Dogs were endotracheally intubated, and anesthesia was maintained with 2% isoflurane in oxygen. Preoperative and postoperative analgesia was achieved with metamizole (50 mg/kg, PO) and methadone (0.1 mg/kg, IV). The CO_2_ was measured using side stream capnography from the endotracheal tube. Animals were ventilated to maintain Pa CO_2_ between 3% and 3.5%. During anesthesia, systolic arterial blood pressure was measured with an oscillometric blood pressure monitor (Pulse Ox/NIBP 6004‐SurgiVet). Heart rate and body temperature were monitored continuously.

### 2.4 IVP measurement

Animals were placed in right lateral recumbency without elevation of the head. A 0.7‐cm diameter craniotomy was drilled by a pneumatic drill (ELAN 4 Aesculap, Braun Vet Care GmbH, Tuttlingen, Germany) into the left parietal bone in each animal. The vessels of the dura mater were cauterized, and a blade incision (11 blade) was performed to open the dura mater. The IVP was measured using a commercially available system for humans (CODMAN ICP EXPRESS Monitor). A single‐use piezoresistive strain gauge sensor mounted in a miniature titanium case at the tip of a flexible nylon catheter (MicroSensor ICP probe) was inserted into the left lateral ventricle for measurement before implantation of the ventricular catheter. Pressure at the tip of the device results in an electrical voltage. The IVP in each dog was measured with a new individual pressure probe which was calibrated in sterile saline before use. A single measurement was performed for each animal. After removing the device, VPS placement was routinely performed using commercially available shunt systems (miniNAV and paediGav Miethke GmbH & Co KG, Potsdam, Germany).

### 2.5 Statistical analysis

Statistical analysis was performed using a commercial statistical software package (Base SAS 9.4 Procedures Guide: Statistical Procedures, 2nd edition ed. Statistical Analysis System Institute Inc., Cary, North Carolina, USA). Because of a large variety of breeds, they were summarized in small breed (<5 kg), medium breed (6‐20 kg), and large breed (>20 kg) to allow statistical evaluation of small, medium, and large breeds. Breed, body weight, and age were evaluated as independent variables. Duration of clinical sings and clinical signs such as an abnormal skull conformation, acoustic deficits, ataxia, behavioral changes (eg, circling, vocalization, aggression, hiding), disorientation, generalized tremor, head tilt, nystagmus, pain (cervical or diffuse), obtundation, seizures and vision deficits were evaluated. The MRI signs such as VBR, corpus callosal height, number of affected ventricles, deformation of the interthalamic adhesion, periventricular edema, disruption of the internal capsule, dilation of the olfactory recess, flattening of gyri and sulci, and susceptibility artifact in T2‐weighted images within the 3rd ventricle or mesencephalic aqueduct (signal void sign) were also evaluated. The presence of an IVP above 12 mm Hg and the presence of SM were evaluated as categorial variables. A Saphiro‐Wilk test was used to test data for normal distribution. A log10 transformation was performed on all variables that were not normally distributed. All clinical and radiological variables were tested individually. Logistic regression analyses were performed to provide an estimate of the strength of association between clinical and radiological variables and the presence or absence of SM, controlling for the confounding effect of the dogs age. Seizures, acoustic impairment, and tremor had a low number of observations, resulting in a complete separation of data points; they therefore had to be excluded from further analysis. For all statistical tests, a significance level of .05 was applied.

Approval from the ethics committee of the Justus‐Liebig‐University was not sought as retrospective studies of images and records stored in the archive are not subject to ethical review.

## 3 RESULTS

### 3.1 Animals

Fifty‐three dogs were included in the study. Median age was 6 months (1‐54 months), with a median body weight of 3.8 kg (1.1‐30 kg). Overall, 25 small breeds (<5 kg), 16 medium breeds (6‐20 kg), and 12 large breeds (>20 kg) were included. There were 26 intact females, 1 neutered female, 23 intact males, and 3 neutered males.

### 3.2 Clinical findings and IVP

All examined dogs experienced clinical signs and neuroanatomical localization was either forebrain (14/53) or multifocal (39/53). The median duration of clinical signs was 36.5 days (5‐672 days). The duration of clinical signs was not associated with an increased IVP (*P* > .05). Clinical signs included cerebello‐vestibular ataxia (29/53), vision deficits (29/53), obtundation (19/53), diffuse or cervical pain (18/53), fixed ventrolateral strabismus (16/53), abnormal skull conformation (12/53), behavioral abnormalities (13/53), disorientation (10/53), positional or spontaneous nystagmus (8/53), head tilt (7/53), seizures (4/53), acoustic impairment (3/53), and generalized tremor (1/53). Disorientation was defined as reduced orientation within the room or to auditory stimuli. Median IVP was 10 mm Hg and ranged from 1 to 35 mm Hg. The IVP was >12 mm Hg in 18 animals. A significant association between IVP and obtundation (*P* = .02), IVP and head tilt (*P* = .04) and between IVP and nystagmus (*P* = .02) was found. The adjusted odds of raised IVP were increased by a factor of 4.64 (95% CI 1.27 to 16.93) for dogs with obtundation compared with those without obtundation. The odds of raised IVP were increased by a factor 6.42 (95% CI 1.08 to 37.97) for dogs with head tilt compared with those without head tilt. The odds of raised IVP was increased by a factor 8.24 (95% CI 1.44 to 47.07) for dogs with nystagmus compared with those without nystagmus (Table 1). Seizures, acoustic impairment, and tremor were not analyzed because of the low number of observations. Other variables were not significantly associated with increased IVP values (*P* > .05).

**TABLE 1:** Summary of explanatory variables and number of observations listed in total and according to IVP measurements > or <12 mm Hg.

Explanatory variable	IVP measurements		Regression coefficient (SE)	Wald Chi‐Square test	*P*‐value	Odds ratio (95% CI)
	<12 mm Hg	>12 mm Hg				
Dogs (total)	35	18				
Signalement						
Breed (<5 kg) vs (>20 kg)	19	6	−4817 (0.4187)	0.42	.25	0.68 (0.15‐3.15)
Breed (6‐20 kg) vs (>20 kg)	8	8	0.5789 (0.4256)	0.43	.17	1.97 (0.134‐2.88)
Breed (>20 kg)	8	4				
Female	17	10	−0.1655 (0.296)	0.31	.58	0.72 (0.23‐2.29)
Male	18	8				
Age (months) log10	12.5	7.6	−0.691 (0.7378)	0.88	.35	0.5 (0.12‐2.13)
Body weight (kg) log10	7.3	8.9	0.3566 (0.6778)	0.28	.6	1.43 (0.38‐5.39)
Clinical signs						
Duration of clinical signs (days) log10	104.4	89.7	−0.2624 (0.5504)	0.23	.633	0.77 (0.26‐2.26)
Ataxia	16	13	1.1585 (0.6334)	3.34	.07	3.19 (0.92‐11.02)
Vision deficits	16	13	1.0661 (0.6329)	2.84	.09	2.9 (0.84‐10.04)
Obtundation	9	10	1.5354 (0.6601)	5.41	**.02**	4.64 (1.27‐16.93)
Pain	10	8	0.8081 (0.6231)	1.68	.19	2.24 (0.66‐7.61)
Strabismus	11	5	−0.2124 (0.6452)	0.11	.74	0.81 (0.23‐2.86)
Skull conformation	8	4	−0.2569 (0.7337)	0.12	.72	0.77 (0.18‐3.26)
Behavioral changes	9	4	−0.1241 (0.6955)	0.03	.86	0.88 (0.23‐3.45)
Disorientation	5	5	0.8095 (0.7184)	1.27	.26	2.25 (0.55‐9.19)
Head tilt	2	5	1.8586 (0.9073)	4.2	**.04**	6.42 (1.08‐37.97)
Nystagmus	2	6	2.1092 (0.889)	5.63	**.02**	8.24 (1.44‐47.07)
MRI findings						
Biventricular Hydrocephalus	4	0	1.0466 (0.5477)	3.65	**.05**	2.85 (0.97‐8.33)
Triventricular Hydrocephalus	18	7				
Tetraventricular Hydrocephalus	13	11				
Periventricular edema	9	16	3.1968 (0.8593)	13.84	**<.0002**	24.46 (4.54‐131.77)
Interthalamic adhesion	30	16	0.1349 (0.9134)	0.02	.88	1.14 (0.19‐6.86)
Internal capsule	24	12	−0.2952 (0.656)	0.2	.65	0.74 (0.21‐2.69)
Olfactory recess	25	12	−0.2623 (0.6324)	0.17	.68	0.77 (0.22‐2.66)
Gyri and sulci	13	10	0.6571 (0.6041)	1.18	.28	1.93 (0.59‐6.3)
Signal void	5	13	2.8529 (0.7469)	14.59	**<.0001**	17.34 (4.01‐74.95)
Syringomyelia	10	9	1.2146 (0.657)	3.42	.06	3.37 (0.93‐12.21)
Ventricle brain ratio (VBR) (unit = 0.1)	0.79	0.77	−4.3466 (3.7421)	1.35	.25	0.65 (0.31‐1.35)
Corpus callosal height (mm) (log10)	13.6	13.3	−1.0887 (1.6229)	0.45	.50	0.34 (0.01‐8.1)

*Note*: Results of logistic regression coefficient and according standard error, Wald Chi‐Square test, *P*‐value and age adjusted odds ratios with confidence interval of 95% (95% CI). Significant *p*‐values are shown in bold.

### 3.3 MRI findings and IVP

The median VBR was 0.77 (0.63‐0.93), and median corpus callosum height was 12 (6.2‐33.7). Most animals were affected by triventricular hydrocephalus (25/53), followed by tetraventricular hydrocephalus (24/53) and biventricular hydrocephalus (4/53). The number of affected ventricles showed an association with an increased IVP (*P* = .05). The adjusted odds of raised IVP were increased by a factor of OR of 2.85 (95% CI 0.97 to 8.33) for dogs with tetraventricular hydrocephalus compared with those with biventricular hydrocephalus (Table 1). A tetraventricular hydrocephalus was associated with the highest risk for a high IVP. Eleven animals with tetraventricular hydrocephalus showed an elevated IVP (>12 mm Hg), and 13 animals showed an IVP of 12 mm Hg or less.

Compared with dogs without periventricular edema, the odds of high IVP for dogs with periventricular edema was increased by a factor of 24.46 (95% CI 4.54 to 131.77) (*P* < .0002). Sixteen animals with periventricular edema had a high IVP. However, 9 animals with periventricular edema did not show high IVP levels (Table 1). Two animals with high IVP values of 13 and 24 mm Hg did not show periventricular edema on MRI.

The presence of T2 signal void sign (18/53) within the 3rd ventricle or mesencephalic aqueduct was also significantly associated with a high IVP (*P* < .0001). The adjusted odds of high IVP were increased by a factor of 17.34 (95% CI 4.01 to 74.95) for dogs with signal void sign compared with those without signal void sign. Overall, 13 animals presented with a signal void sign and high IVP, whereas 5 animals with signal void showed normal IVP values. Five animals with high IVP values did not show a signal void sign.

Deformation of the interthalamic adhesion (46/53), dilation of the olfactory recess (37/53), disruption of the internal capsule (36/53), flattening of gyri and sulci (23/53), and SM (19/53) were not significantly associated with a high IVP (*P* > .05) (Table 1).

### 3.4 Syringomyelia

Increasing age, was associated with SM, with an OR of 4.28 (95% CI 0.99 to 18.43) (*P* = .05). The number of affected ventricles was significantly associated with the presence of SM, with an OR of 12.74 (95% CI 2.93 to 55.4) (*P* = .0007). Increasing numbers of affected ventricles increased the probability of SM, with tetraventricular hydrocephalus being at highest risk (Table 2). A lower VBR was associated with the presence of SM (*P* = .004). An increasing VBR by 0.1 decreased the odds of SM by a factor of 0.25 (95% CI 0.1 to 0.63). The presence of SM was also significantly increased in cases with periventricular edema (*P* = .004), and signal void sign (*P* = .03). The adjusted odds of SM were increased by a factor of 7.61 (95% CI 1.91 to 30.32) for dogs with periventricular edema compared with those without periventricular edema. The odds of SM were increased by a factor 4.18 (95% CI 1.16 to 15.02) for dogs with signal void sign compared with those without signal void sign. Another significant association was found for the disruption of the internal capsule, with an OR of 0.19 (95% CI 0.05 to 0.72) (*P* = .01). In dogs with disruption of the internal capsule, the odds of SM were decreased compared to those with intact internal capsule. The other MRI findings did not reveal a significant association with the presence of SM (*P* > .05). Pain was significantly associated to the presence of SM (*P* = .05). The odds of showing pain were increased by a factor of 3.4 (95% CI 0.98 to 11.78) in dogs with SM compared to dogs without SM. No other clinical signs were associated with SM in the study population (*P* > .05) (Table 2).

**TABLE 2:** Summary of explanatory variables and number of observations listed in total and according to the presence of syringomyelia.

Explanatory variable	Syrinx		Regression coefficient (SE)	Wald Chi‐Square test	*P*‐value	Odds ratio (95% CI)
	No	Yes				
Dogs (total)	34	19				
Signalement						
Breed (<5 kg) vs (>20 kg)	16	9	−0.2198 (0.4236)	0.27	.6	0.83 (0.18‐3.88)
Breed (6‐20 kg) vs (>20 kg)	10	6	0.2486 (0.4502)	0.3	.58	1.32 (0.26‐6.75)
Breed (>20 kg)	8	4				
Female	15	12	−0.4006 (0.308)	1.69	.19	0.45 (0.13‐1.5)
Male	19	7				
Age (mo)	7.9	16.1	1.4528 (0.7454)	3.8	**.05**	4.28 (0.99‐18.43)
Body weight (kg)	7.4	8.8	0.3602 (0.6813)	0.28	.6	1.43 (0.38‐5.45)
Clinical signs						
Duration of clinical signs (days) log_10	109.6	78.1	−1.0739 (0.6315)	2.89	.09	0.34 (0.1‐1.18)
Ataxia	17	12	0.5656 (0.6111)	0.86	.35	1.76 (0.53‐5.83)
Vision deficits	18	11	0.4959 (0.631)	0.62	.43	1.6 (0.48‐5.66)
Obtuntation	11	8	0.2037 (0.6235)	0.11	.74	1.23 (0.36‐4.16)
Pain	8	10	1.2249 (0.6334)	3.74	**.05**	3.4 (0.98‐11.78)
Strabismus	9	7	0.6288 (0.6439)	0.95	.33	1.88 (0.53‐6.62)
Skull conformation	8	4	0.3216 (0.7577)	0.18	.67	1.3 (0.31‐6.09)
Behavioral changes	10	3	−1.0501 (0.7808)	1.81	.18	0.35 (0.08‐1.62)
Disorientation	8	2	−0.8914 (0.8665)	1.06	.3	0.41 (0.08‐2.24)
Head tilt	4	3	0.4239 (0.8597)	0.24	.62	1.53 (0.28‐8.24)
Nystagmus	5	3	0.1793 (0.8191)	0.05	.83	1.2 (0.24‐5.96)
MRI findings						
Biventricular Hydrocephalus	4	0	2.545 (0.7498)	11.52	**.0007**	12.74 (2.93‐55.4)
Triventricular Hydrocephalus	21	4				
Tetraventricular Hydrocephalus	9	15				
Periventricular edema	11	14	2.0293 (0.7054)	8.28	**.004**	7.61 (1.91‐30.32)
Interthalamic adhesion	30	16	−0.0287 (0.8742)	0.001	.97	0.97 (0.18‐5.39)
Internal capsule	28	8	−1.6519 (0.6716)	6.05	**.01**	0.19 (0.05‐0.72)
Olfactory recess	25	12	−0.4303 (0.642)	0.45	.5	0.65 (0.19‐2.29)
Gyri and sulci	17	6	−0.5392 (0.6289)	0.74	.39	0.58 (0.17‐2.0)
Signal void	8	10	1.4308 (0.6525)	4.81	**.03**	4.18 (1.16‐15.02)
Ventricle brain ratio (VBR) (unit = 0.1)	0.82	0.73	−13.854 (4.7421)	8.54	**.004**	0.25 (0.1‐0.63)
Corpus callosal height (mm)	14.3	12.3	−0.8537 (1.6163)	0.28	.6	0.43 (0.02‐10.12)

*Note*: Results of logistic regression coefficient and according SE, Wald Chi‐Square test, *P*‐value and age adjusted odds ratios with confidence interval of 95% (95% CI). Significant *p*‐values are shown in bold.

## 4 DISCUSSION

The most common clinical signs reported in animals with internal communicating hydrocephalus are visual impairment, obtundation, ataxia, behavioral changes, and strabismus, as well as head tilt and nystagmus.^1^, ^20^, ^21^, ^22^, ^23^ This is consistent with the most reported clinical findings in our study. Seizures are also reported with a low prevalence, which is in agreement with our observations.^24^ A high IVP was associated with obtundation, head tilt and nystagmus in this study. Obtundation is an abnormal mentation reflecting the involvement of the 4th ventricle and compression of the brainstem with subsequent impairment of the ascending reticular activating system or limbic components of the cerebrum and diffuse forebrain involvement.^25^, ^26^, ^27^ Vestibular signs are associated with dilation of the 4th ventricle.^1^, ^20^, ^21^, ^22^, ^23^ This is in partial agreement with our observations, although not all cases with tetraventricular hydrocephalus showed vestibular signs. The degree of 4th ventricle enlargement might play a role in the development of vestibular signs, but this has never been investigated. Nystagmus was observed in 8 animals of which 5 had dilation of all 4 ventricles, while 3 had a triventricular hydrocephalus. Reduced cerebral white matter volume and perfusion occur in animals with internal hydrocephalus.^28^, ^29^, ^30^ These changes might also occur at the level of the brainstem and midbrain in case of tetraventricular hydrocephalus and at the level of the thalamus in cases of triventricular hydrocephalus resulting in vestibular clinical signs, but this remains speculative.

Periventricular hyperintensities on T2 and FLAIR images, consistent with interstitial edema, were significantly correlated with elevated IVP. It was suggested that if the IVP exceeds a critical level, the ependymal lining is damaged and CSF leaks from the ventricles into the brain parenchyma, producing periventricular interstitial edema.^31^ However, some animals in our study did show periventricular edema without an elevation of IVP. The absence of an elevated IVP at the time of measurement might be because of the elastic and adaptive properties of the brain parenchyma, which yields under the expansion of the ventricles, resulting in IVP decline. There are no data concerning the necessary timespan for periventricular oedema to resolve, which might linger on for a certain time after IVP normalization. Furthermore, the measurements were performed under general anesthesia, and the IVP might therefore be even higher in the awake animal.^4^ Although periventricular edema might not represent an acute high IVP and a life‐threatening situation, it might result in white matter inflammation, ischemia, demyelination, gliosis, and glial scarring if not corrected rapidly, which again indicates near‐term surgical intervention.^32^, ^33^ Periventricular edema is related to clinical signs such as obtundation.^16^ This close connection of clinical and MRI findings suggests periventricular edema as a valuable tool for clinical assessment and decision‐making regarding VPS treatment and selection of an appropriate pressure‐valve system.

A signal void sign, which is observed as a T2‐hypointense CSF signal within the ventricular spaces because of high flow velocities, was also significantly associated with an increased IVP. The signal‐void sign is related to CSF flow and turbulence during MR image acquisition.^34^ The underlying mechanism of this phenomenon is attributed to an increase of the pulse pressure of the arterial flow within the brain parenchyma, which is transmitted to the CSF flow through pulsation of the choroid plexus.^35^ It can occur in animals with a normal brain but is a common finding in hydrocephalic dogs.^34^ While there is debate about the dynamics of CSF in hydrocephalic dogs, the presence of a signal void sign was explained by a hyperdynamic CSF flow from the lateral ventricles through the aqueduct at a higher velocity.^35^

The association between IVP and signal void sign in this study was clear, although not all dogs with raised IVPs also showed a signal void sign, which might have various reasons. Technical variations (slice thickness, length of echo time) are unlikely as all dogs were scanned under the same conditions in our hospital, although magnetic field strength varied between 1.5 and 3.0 Tesla, which should not influence the occurrence of a signal void. As CSF hydrodynamics is intimately associated with the cardiac cycle, a physiologic reason for an altered CSF flow is an altered heart rate under general anesthesia in each individual dog, which might result in differences among the presence of a signal void.^36^

The number of affected ventricles, especially the presence of tetraventricular hydrocephalus, was significantly associated with an increased IVP. The pathophysiological development of congenital tri‐, or tetraventricular hydrocephalus is unclear in most cases. An obliteration of the lateral apertures might occur because of inflammation of the choroid plexus of the 4th ventricle.^37^, ^38^ Changes in the choroid plexus rather cause acute hydrocephalus by a sudden block of the CSF outflow and therefore might result in raised IVP.^37^, ^38^ In experimental hydrocephalus, hypersecretion from the choroid plexus following intraventricular hemorrhage was observed, which might clear pathogens or debris from the epithelial surface.^39^, ^40^, ^41^ This effect might contribute to ventricular enlargement and internal hydrocephalus with raised IVP. Other explanations for the occurrence of tetra‐ or triventricular hydrocephalus might be impaired regional cerebral perfusion, impaired lymphatic drainage, and build‐up of toxic metabolites in these regions, which occur in humans with internal hydrocephalus and CSF flow disturbances in the caudal cranial fossa and the craniocervical junction, with similar descriptions in dogs.^28^, ^42^, ^43^, ^44^, ^45^, ^46^, ^47^

Some dogs with internal hydrocephalus also exhibit SM.^3^ Placement of a IVP Shunt might reduce SM in animals presented with both hydrocephalus and SM.^3^ Animals with tetraventricular hydrocephalus and a signal void sign were also most likely to show concurrent SM. Usually, abnormalities of the craniocervical junction are associated with SM, especially in small‐breed dogs.^46^, ^47^ Some dogs in our study population included in the small‐breed group might have been affected by craniocervical junction abnormalities as well, but this does not explain our findings as no association between small, medium, or large breeds and the presence of SM were found here. Increasing age and pain was associated to the presence of SM in this study, which is in agreement with the findings of another study in which SM was associated with increasing age.^48^, ^49^ The disruption of the internal capsule, in contrast, decreased the probability of concurrent SM. There is no definitive explanation for this finding. A disruption of the internal capsule might be found in higher grades of ventricular enlargement as it is proceeded by an atrophy of white matter.^29^ As a result, the CSF space becomes greater, and CSF flow disturbances might become less severe and relevant. A lower VBR is also associated with SM, which would contribute to this hypothesis.

VPS offers a macroscopical reconstitution of the brain parenchyma and resolution of clinical signs in both hyper‐ and normotensive internal hydrocephalus in humans and animals.^16^, ^50^, ^51^, ^52^ Diagnosis of internal hydrocephalus with normal IVP and recommendation for VPS implantation in humans is based on the Hakims triad of clinical signs (gait disturbance, incontinence, and mental deterioration), combined with an Evans Index >0.3 in MRI, which resembles the here measured VBR.^50^, ^53^ Such combination of clinical signs and MRI findings needs to be established for the diagnosis of internal hydrocephalus in animals as well. In this study, however, the aim was to identify clinical signs and MRI findings indicative for elevated IVP. In human literature uncontrolled increased ICP in the context of hydrocephalus can result in nausea, vomiting, headaches, vision loss, and, if left untreated, death.^10^, ^11^, ^54^ These cases therefore require a more urgent VPS treatment compared to those without increased IVPs, a similar recommendation might be given for animals as well.^7^, ^8^, ^9^, ^10^, ^11^, ^54^ However further evaluation of the severity of clinical signs and prognosis in case of raised IVP in congenital hydrocephalus in animals is warranted to make a proper recommendation for treatment.

Key imaging features are recommended in humans to predict outcomes after VPS treatment in patients with internal hydrocephalus. These include ventricular enlargement (Evans Index >0.3) and one of the following: (A) Enlargement of the temporal horns of the lateral ventricles not entirely attributable to hippocampus atrophy; (B) corpus callosal angle >40°; (C) periventricular changes; (D) signal void within the ventricular system.^50^ These MRI signs are routinely evaluated in canine and feline hydrocephalic cases.^55^, ^56^ In this study, we prove an association with raised IVP measurements for some of these variables, but further studies are needed to assess their predictive values for outcomes after VPS treatment in animals.

This study has some limitations, first of all given its retrospective character and the low number of some clinical signs, especially seizures, tremor, and acoustic impairment. Seizures are a rare finding in animals with internal hydrocephalus, which explains our low number of observations.^24^ Brainstem auditory evoked potentials could be performed to increase information about acoustic function in these animals as clinical examination remains rather subjective and based on owner reports. The low sample size is also reflected by large ranges within the confidence intervals of 95% of some variables. A larger sample size would help improve the statistical power of our results and to further evaluate some of the fewer observed symptoms. Some measurement errors with regard to morphometric MRI variables, such as VBR and corpus callosal height, cannot be completely excluded. Given the retrospective character of the study, slice thickness and MRI planes might have differed a few millimeters among the cases, which might have resulted in mild differences within the performed morphometric measurements (VBR and corpus callosal height), which were all performed at the same anatomic level. Another limitation is the lack of an outcome evaluation for these clinical and MRI findings as this might give some guidelines regarding treatment. Furthermore, only single IVP measurements were performed under general anesthesia during surgery. This does not properly reflect the pressure dynamics of an awake dog and higher pressures must be expected in awake animals.^4^ Telemetric IVP measurements in animals with internal hydrocephalus would provide more information about CSF flow dynamics and could improve our understanding of the pathophysiology of internal hydrocephalus in animals.

## 5 CONCLUSION

The presence of tetraventricular hydrocephalus, periventricular edema, and signal void sign is associated with a high IVP. Obtundation, head tilt and nystagmus are clinical signs indicative of raised IVPs in dogs. Other previously reported MRI findings might not be suitable to diagnose raised IVPs. Clinical signs and MRI findings should be used to make a diagnosis of internal hydrocephalus with or without increased IVP.

## CONFLICT OF INTEREST DECLARATION

Authors declare no conflict of interest.

## OFF‐LABEL ANTIMICROBIAL DECLARATION

Authors declare no off‐label use of antimicrobials.

## INSTITUTIONAL ANIMAL CARE AND USE COMMITTEE (IACUC) OR OTHER APPROVAL DECLARATION

Authors declare no IACUC or other approval was needed.

## HUMAN ETHICS APPROVAL DECLARATION

Authors declare human ethics approval was not needed for this study.
